# Predictors of treatment REsponse to inhaled corticosteroids (ICS) in Chronic Obstructive pulmonary disease: randomised controlled trials individual participant Data re-Evaluation–protocol of the ICS-RECODE individual participant data meta-analysis

**DOI:** 10.1136/bmjopen-2024-095541

**Published:** 2025-03-05

**Authors:** Sebastian Bate, Rebecca Fortescue, Catherine Fullwood, Matthew Sperrin, Mark Simmonds, Markus Fally, Jan Hansel, Michael Miligkos, Sinduja Manohar, Emily Howlett, John Linnell, Alan Preston, the ICS-RECODE group, Helen Ashdown, Ashley A Woodcock, Dave Singh, Lesley Stewart, Joergen Vestbo, Alexander G Mathioudakis

**Affiliations:** 1 Research and Innovation, Manchester University NHS Foundation Trust, Manchester, UK; 2 Centre for Biostatistics, The University of Manchester, Manchester, UK; 3 Population Health Research Institute, St George’s University of London, London, UK; 4 Centre for Health Informatics, Imaging and Data Science, Faculty of Biology, Medicine and Health, The University of Manchester, Manchester Academic Health Science Centre, Manchester, UK; 5 Centre for Reviews and Dissemination, University of York, York, UK; 6 Department of Respiratory Medicine and Infectious Diseases, Copenhagen University Hospital—Bispebjerg and Frederiksberg, Copenhagen University Hospital, Kobenhavn, Denmark; 7 Division of Immunology, Immunity to Infection and Respiratory Medicine, Faculty of Biology, Medicine and Health, The University of Manchester, Manchester, UK; 8 North West School of Intensive Care Medicine, Health Education England North West, Manchester, UK; 9 Allergy Department, 2nd Paediatric Clinic, National and Kapodistrian University of Athens, Athens, Greece; 10 Vocal, Manchester University NHS Foundation Trust, Manchester, UK; 11 COPD Foundation, Miami, Florida, USA; 12 North West Lung Centre, Wythenshawe Hospital, Manchester University NHS Foundation Trust, Manchester Academic Health Science Centre, Manchester, UK; 13 Medicines Evaluation Unit, Manchester, UK; 14 Department of Internal Medicine, Herlev Gentofte University Hospital, Section of Respiratory Medicine, Copenhagen University Hospital, Kobenhavn, Denmark

**Keywords:** Pulmonary Disease, Chronic Obstructive, Randomized Controlled Trial, Meta-Analysis, Systematic Review

## Abstract

**Abstract:**

**Introduction:**

Inhaled corticosteroids (ICS) can improve clinical outcomes in patients with chronic obstructive pulmonary disease (COPD) and eosinophilic airway inflammation, but they also increase the risk of side effects like pneumonia. Blood eosinophils guide ICS use, though evidence is limited. The predictors of treatment REsponse to ICS in COPD: a randomised controlled trials (RCTs) individual participant Data re-Evaluation (ICS-RECODE) research programme will leverage data from large RCTs to identify patients who benefit most from ICS with minimal risk. This protocol details an individual participant data (IPD) meta-analysis, assessing ICS safety, efficacy and treatment×covariate interactions to identify predictors of treatment response.

**Methods and analysis:**

This meta-analysis will adhere to Cochrane, IPD handbook and Grading of Recommendations Assessment, Development and Evaluation (GRADE) guidance. We will conduct a two-stage IPD meta-analysis of RCTs evaluating the addition of ICS to maintenance COPD treatments. Only RCTs with at least 500 participants across all eligible arms will be included, to allow for treatment×covariate interaction evaluation. Primary outcomes are severe and moderate or severe exacerbation rates; secondary outcomes assess both safety and efficacy. Data from each RCT will be reanalysed using rigorous, consistent statistical methods. Treatment×covariate interactions will be assessed at the RCT level. Trial treatment effects and the coefficients of treatment×covariate interaction analyses will be pooled using random effects model meta-analysis. Risk of bias will be appraised using RoB-2 informed by IPD, and certainty of evidence will be assessed with GRADE and the Instrument to assess the Credibility of Effect Modification Analyses.

The ICS-RECODE IPD meta-analysis will make use of the best available data to define evidence-based, precision medicine approaches for ICS use in COPD.

**Ethics and dissemination:**

The Health Research Authority approved the ICS-RECODE study, exempting it from ethics review (HRA UK, Reference: 24/HRA/0460). Our findings will be published in peer-reviewed journals and shared with the scientific and broader stakeholder communities.

**PROSPERO registration number:**

CRD42024508286.

**STRENGTHS AND LIMITATIONS OF THIS STUDY**

- High-quality data from 22 randomised controlled trials (RCTs), encompassing >50 000 participants to assess the safety, efficacy and predictors of treatment response to inhaled corticosteroids in chronic obstructive pulmonary disease.
- Rigorous, consistent and prospectively planned methodology for reanalysing the RCTs and pooling their data.
- Multistakeholder input, including consistent engagement of patients and the public.
- Strict eligibility criteria and explanatory design of the included RCTs may limit the generalisability of our findings, which will need to be validated in a real-life setting.

## Introduction

 Chronic obstructive pulmonary disease (COPD), a leading cause of death and disability globally,^1 2^ is characterised by marked heterogeneity in both clinical manifestations and underlying mechanisms, thus representing a prime target for the introduction of precision medicine interventions.^3 4^ Characteristically, inhaled corticosteroids (ICS) appear to be effective only for patients with enhanced airway eosinophilic inflammation.^3 4^ In these patients, they reduce the frequency of exacerbations, improve quality of life, decelerate lung function decline and possibly reduce mortality.^5^,^8^ However, these benefits come at the expense of side effects that include a significant increase in the risk of pneumonia.^9 10^ This is a concerning risk, since the 6-month mortality after a hospital admission for pneumonia versus exacerbation without pneumonia was recently estimated to be 20% and 3%, respectively, among patients with COPD.^11^ Importantly, a recent analysis suggested that the excess risk of pneumonia may be confined to patients who do not benefit from ICS.^12^

Blood eosinophil count (BEC) is used to guide ICS administration for COPD.^13^ However, in recent guidelines, both the American Thoracic Society and National Institute of Health and Care Excellence (UK) found only weak evidence supporting the use of BEC or other biomarkers to target ICS administration.^14 15^ Both organisations prioritised relevant research.^14 15^ Moreover, other parameters, such as smoking status, may be associated with treatment response to ICS in COPD.^16 17^

The ICS-RECODE (predictors of treatment REsponse to ICS in COPD: a randomised controlled trials (RCTs) individual participant Data re-Evaluation) individual participant data (IPD) meta-analysis will make use of the best available evidence from large, well-conducted RCTs to identify patients with COPD who will gain most benefit from the administration of ICS, at the lowest risk of severe side effects. Here, we present the methods for the pivotal part of the ICS-RECODE study, an IPD meta-analysis evaluating the safety and efficacy of ICS for COPD and exploring potential treatment×covariate interactions for prospectively selected biomarkers and clinical variables (to identify whether particular patient subgroups benefit more or less from ICS).

## Methods and analysis

**Figure 1:**
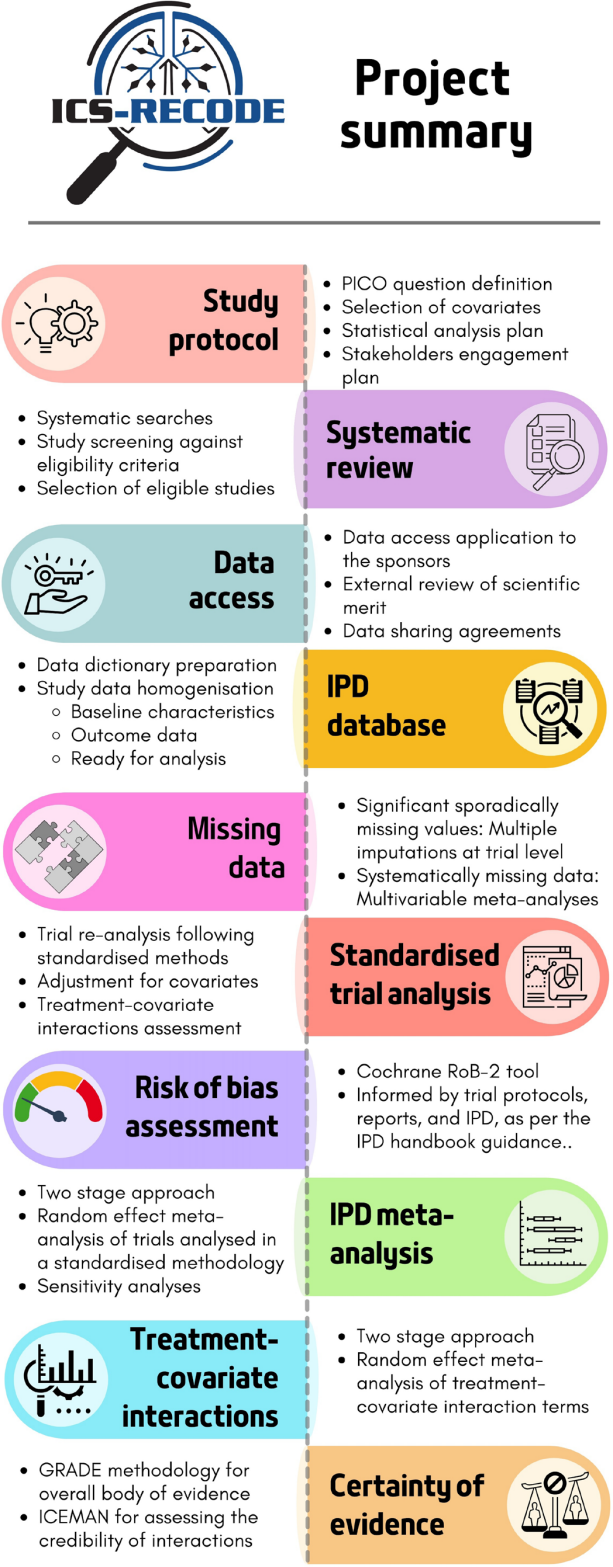
The ICS-RECODE study summary. ICS-RECODE, predictors of treatment REsponse to inhaled corticosteroids (ICS) in Chronic Obstructive pulmonary disease: randomised controlled trials individual participant Data re-Evaluation; ICEMAN, Instrument to assess the Credibility of Effect Modification Analyses; IPD, individual participant data; GRADE, Grading of Recommendations Assessment, Development and Evaluation.

This IPD meta-analysis has been prospectively registered with PROSPERO (ID: CRD42024508286).^18^ It will be conducted following guidance by Cochrane,^19^ the Grading of Recommendations Assessment, Development and Evaluation (GRADE) Working Group^20^ and the IPD handbook.^21^ It will be reported following the Preferred Reporting Items for Systematic Reviews and Meta-Analyses (PRISMA) extension for IPD meta-analyses.^22^ This protocol is produced in line with the PRISMA-P^23^ reporting guidance for systematic review protocols. Stakeholder engagement will be reported following the “Be ACTIVE” (ACTIVE) framework.^24^ The study methodology is summarised in figure 1.

### Eligibility criteria

We will consider RCTs evaluating maintenance management in COPD of any severity, provided that the COPD diagnosis was based on standard clinical and lung function criteria and that the RCT reports on the baseline spirometric severity and exacerbations history. More specifically, we will include studies evaluating ICS as a maintenance management for COPD, as a monocomponent, or as part of an established combination of inhaled medications. We will accept studies comparing ICS with a corresponding non-ICS-containing combination (ie, long-acting beta-2 agonists - LABA/ long-acting muscarining antagonists - LAMA/ICS vs LABA/LAMA; LABA/ICS vs LABA; ICS vs placebo). The administration of any concomitant COPD treatments will be permitted, provided they are not part of the randomised intervention. We will select studies with an overall, relevant study population of at least 500 participants. This threshold was pragmatically selected to ensure sufficient power for treatment×covariate interactions. We will exclude studies that do not assess any of the outcomes of interest and those that do not report on the baseline exacerbation rate prior to recruitment. At a study participant level, we will exclude those with concomitant a1-antitrypsin deficiency, those receiving biologic treatments for their airway disease, and those who did not receive any doses of the study treatment.

### Systematic searches

As required for IPD meta-analyses, searches and study identification were completed before the protocol was finalised to estimate the necessary resources and initiate the data access process. We searched the Cochrane Central Register of Controlled Trials (CENTRAL) and the Cochrane Airways Trials Register (CATR), which capture RCTs and systematic reviews from all major online libraries, including (but not limited to) Medline, EMBASE, CINAHL and the WHO’s International Clinical Trials Registry Platform. We used a structured search strategy including both dictionary and free search terms to look for RCTs and meta-analyses evaluating ICS for COPD (box 1). This yielded 2585 titles (updated: October 2024). Two investigators independently assessed all identified studies, including the reference lists of relevant systematic reviews, for eligibility at a title/abstract level, followed by a full-text evaluation of all potentially eligible studies. There were no disagreements in the study selection. We captured eligible ongoing and completed RCTs. We identified 31 eligible completed RCTs totalling >65 000 eligible participants. These are described in online supplemental table S1.

Box 1 **Search strategy**

((Pulmonary Disease, Chronic Obstructive [MH]) or (Lung Diseases, Obstructive [MH:noexp]) or (Emphysema [MH]) or (Bronchitis, Chronic [MH]) or (COPD [tiab]) or (emphysema [tiab]) or (chronic bronchitis [tiab])) and

((Beclomethasone [MH]) or (Budesonide [MH]) or (Fluticasone [MH]) or (Mometasone Furoate [MH]) or (Triamcinolone [MH]) or (Beclomethasone [tiab]) or (Beclometasone [tiab]) or (Budesonide [tiab]) or (Fluticasone [tiab]) or (Ciclesonide [tiab]) or (Mometasone [tiab]) or (Flunisolide [tiab]) or (Triamcinolone [tiab]) or (ICS [tiab]) or (Trimbow [tiab]) or (Trelegy [tiab]) or (Trixeo [tiab]) or (Symbicort [tiab]) or (Dulera [tiab]) or (Breo [tiab]) or (Airduo [tiab]) or (Advair [tiab]) or (Seretide [tiab]) or (Duoresp [tiab]) or (Flutiform [tiab]) or (Fostair [tiab]) or (Relvar [tiab]) or (Sirdupla [tiab]) or (Viani [tiab]) or (Qvar [tiab]) or (Flovent [tiab]) or (Alvesco [tiab]) or (Asmanex [tiab]) or (Flixotide [tiab]) or (Arnuity [tiab]) or (Pulmicort [tiab]) or (Aerospan [tiab]) or (Aerobid [tiab]) or (Beclovent [tiab]) or (AmronAir [tiab])) not

((Child [MH]) not (adult [MH]))

### Data access and homogenisation

All eligible RCTs were sponsored by the pharmaceutical industry and we have applied for data access, in line with recommendations by the European Federation of Pharmaceutical Industries and Associations and the Pharmaceutical Research and Manufacturers of America and the European Medicines Agency. Applications were made either through one of the following databases: vivli.org, clinicalstudydatarequest.com, or—when that was not possible—by contacting the sponsor directly. We applied for access to the IPD of all eligible studies. As of October 2024, we have gained access to the IPD for most eligible RCTs (see online supplemental table S1). We did not identify any pattern to the trials for which IPD were declined.

Trial-level data management information will be tracked in Microsoft Excel. IPD will be curated and analysed within the sponsor-nominated platforms using the R statistical package (V.4.3 or newer, R core team, Vienna, Austria). We will not gain access to variables that enable identification of participants’ identity.

A data dictionary/schema with detailed definitions of all variables to be used, including baseline characteristics, outcome data and other relevant covariates have been developed and will guide data homogenisation and establish the IPD database structure. The specifications of the data dictionary are based on the Clinical Data Interchange Standards Consortium (CDISC) nomenclature and the dictionary will be locked before any meta-analyses are conducted. Data from the included trials will be reformatted and recoded in line with the dictionary.

### Outcomes

The primary outcomes of this meta-analysis will be:

- Moderate or severe exacerbation rate.
- Severe exacerbation rate.

For the purposes of this work, moderate exacerbations are those treated with systemic corticosteroids and/or antibiotics but do not require hospital admission. Severe exacerbations are those requiring hospital admission. We anticipate variability in the classification of exacerbations leading to emergency department visits, with some trials categorising them as moderate and others as severe. Preferably, data from exacerbation dates, hospital visits and use of non-trial medication (ie, antibiotics and corticosteroids) will be used to determine severity. If this data are not available within the study datasets, trial-reported severity will be used. Mild exacerbations are acute episodes of increased symptoms, which are beyond the normal day-to-day variation, which do not necessitate systemic treatment (antibiotics or corticosteroids) or hospital admission. We will accept patient-reported mild exacerbations as well as those identified through validated questionnaires or patient diaries.

The secondary outcomes will be:

- Mortality: time-to-death.
- Exacerbations: rate of exacerbations of any severity; rate of exacerbations treated with antibiotics only (presumed infective); rate of exacerbations treated with systemic corticosteroids only (presumed non-infective); time-to-first moderate or severe exacerbation; time-to-first severe exacerbation; time-to-first exacerbation of any severity; time-to-first exacerbation treated with antibiotics only (presumed infective); time-to-first exacerbation treated with systemic corticosteroids only (presumed non-infective).
- Pulmonary function: forced expiratory volume in 1 s (FEV_1_); forced vital capacity (FVC).
- Health-related quality of life: St George’s Respiratory Questionnaire, or any other validated instrument used by the included studies.
- Breathlessness: Modified Borg Dyspnoea Scale, modified Medical Research Council Scale (mMRC), Transition Dyspnoea Index or other validated tests for assessing breathlessness.
- Exercise capacity: 6 min walking test, incremental shuttle walk test or any other validated instruments used by the included studies.
- Fatigue: any validated method such as the Fatigue Severity Scale or the Brief Fatigue Inventory.
- Sleep quality: any validated method such as the Insomnia Severity Index, or the Pittsburgh Sleep Quality Index.
- Pneumonia: time-to-first pneumonia diagnosis. We will accept the diagnostic criteria used by each trial for pneumonia.
- Serious adverse events: rate of serious adverse events and time-to-first serious adverse event, in line with the International Council for Harmonisation of Technical Requirements for Pharmaceuticals for Human Use definition.

Secondary outcomes will be treated equally, without hierarchy.

As we will have access to the dates of events such as exacerbations, death or serious adverse events, we will assess those outcomes at up to 6 months and at up to 12 months (we will only include on-treatment study events and we will only include studies with a follow-up period of at least 9 months in the latter timepoint). All other outcomes will be assessed at 5–7 months and at 11–13 months of follow-up.

### Covariates

Treatment×covariate interactions will be assessed for the following covariates: (1) BEC at baseline, (2) BEC measured at baseline, while patients were not receiving any corticosteroids, (3) BEC measured at baseline, while patients were receiving ICS, (4) current or previous diagnosis of asthma or atopy, (5) reversibility of airflow limitation, (6) FEV_1_ variability, (7) diurnal peak expiratory flow rate variation, (8) asthma features as described in National Institute for Health and Care Excellence (NICE) COPD guidelines, (9) smoking status at baseline (current or former) and (10) ICS dose.

In addition, the following prognostic factors will be accounted for in our models: (1) age, (2) gender, (3) smoking status, (4) baseline exacerbation rate, (5) baseline symptoms severity measured preferably with the COPD assessment test or alternatively with the mMRC scale, (6) baseline FEV_1_, (7) concomitant COPD treatments, (8) baseline BEC, (9) reversibility of airflow limitation, (10) predominance of chronic bronchitis versus emphysema, (11) comorbidities, defined as number of affected body systems.

None of the continuous covariates will be dichotomised. The selection of these covariates was informed by a scoping review of relevant literature and consensus among the investigators.

### Data collection

For baseline characteristics, we will use the last relevant observation at, or prior to, baseline. For example, if there are two or more prerandomisation visits, data will preferably be taken from the last one but if it is not collected at the later visit(s), earlier data may be used. In the case of BEC on/off, *on ICS* shall be a dose of ICS within 2 days of the blood test, and *off ICS* shall be no ICS received within 7 days before the blood test. *On/Off ICS* and change can be gleaned from patients with changes to their medication during run-in periods.

### Missing data

We will assume that data are missing at random. If more than 5% of data for a specific outcome are missing, we will address this using multiple imputations at the level of each trial. If missingness is below this threshold, participants with missing outcome data will be excluded from the analysis for that particular outcome. To deal with prognostic factors systematically missing in some trials, we will use multivariate meta-analyses of partially and fully adjusted results, for the primary outcome.^21^

### Analysis plan

We will conduct a two-stage IPD meta-analysis for several reasons: (1) data will be accessed through different online databases and it will not be possible to analyse the IPD from all RCTs using a single dataset; (2) the second stage uses well-known meta-analysis and reporting methods, which readers will be more familiar with, and performs at least as well as the one-stage method; (3) it allows us to avoid aggregation bias by ensuring that only within-trial information is used in the first stage of the analysis, minimising potential analytical bias and (4) two-stage approaches are stronger for assessing treatment×covariate interactions.^21^

In the first stage of the meta-analysis, we will reanalyse all outcomes in each of the included studies, using consistent methodology and accounting for the previously described predefined prognostic factors. Specifically, we will conduct modified intention-to-treat analyses including all participants that fulfil the eligibility criteria and have sufficient analysable data.

We will use generalised regression models for analysing continuous outcomes, and adjust for the outcomes’ baseline values, in addition to the predefined parameters. Negative binomial models will be used for assessing exacerbations, pneumonia and serious adverse events, with offset for time on treatment. Logistic regression will be used for assessing binary data, while for time-to-event outcomes, we will use Cox regression, provided the proportional hazards assumption is reasonably met (based on Schoenfeld residuals) and no significant competing risks are present. Studies with a significant lack of proportionality, despite our adjustments for multiple prognostic factors, will be excluded.

In the second stage, random-effects meta-analysis will be fitted using a restricted maximum likelihood estimation. The Hartung-Knapp-Sidik-Jonkmak approach will be used for calculating CIs. Heterogeneity in all meta-analyses will be summarised by the estimate of between-trial variance of true effects, and we will also report a 95% prediction interval for the potential treatment effect in a new trial.

We will explore interactions between the administration of ICS and any of the predefined covariates (potential effect modifiers), using a two-stage approach to avoid aggregation bias. For each covariate, we will repeat all previously described analyses for each of the outcomes, accounting for preselected covariates (excluding those associated with the index variable), but also including a treatment×covariate interaction term. In the second stage, the interaction terms of individual trials will be pooled in a random effects meta-analysis model, as described above. We will report an overall estimate of the predictive value of covariates, along with their confidence intervals. We will be using the methods suggested in the IPD handbook.^21^

### Sensitivity analyses

We will perform several sensitivity analyses to explore the robustness of our findings and assess the impact of potential biases. Specifically, we will perform the following sensitivity analyses for the primary outcomes:

- Explore whether the addition of aggregate data from trials whose IPD will not be available to us change the overall results.
- Restrict the meta-analysis to trials of low risk of bias.
- Exclude patients with a history of asthma and/or confirmed airway reversibility because asthma is responsive to ICS and we would like to ensure the inclusion of patients with concomitant asthma does not modify/weaken our findings.
- Assess withdrawal effects of baseline inhaled treatments, such as ICS.^25^ To do this, we will assess separately patients who were receiving ICS at baseline, prior to treatment, and were subsequently randomised to continue receiving or withdraw from ICS, and patients who were not receiving ICS at baseline and were subsequently randomised to start or not start ICS.
- Only include studies evaluating a fixed triple combination (LABA+LAMA+ICS) versus the respective dual bronchodilators (LABA+LAMA) because, in these studies, the treatment regimens are more standardised, while the impact on ICS is tested in the currently recommended treatment step (as an add-on treatment two dual bronchodilator).
- Explore differences in treatment effects according to ICS dose (low–medium–high).

### Risk of bias and certainty appraisal

Risk of bias will be assessed using the RoB-2 tool,^26^ and judgements will be informed by trial protocols, reports and IPD as recommended in the IPD meta-analysis handbook (eg, assessment of the random sequence, treatment deviations and missing outcome data).^21^ Risk of bias will be appraised by two investigators independently. Disagreement will be resolved with discussion or adjudication by a third, independent investigator.

Although IPD will already be cleaned by those responsible for the RCT, we will still check the validity, range and consistency of the variables, alongside assessing for potential risk of bias to inform RoB-2 assessments.

We will use the Instrument to assess the Credibility of Effect Modification Analyses tool for assessing the credibility of treatment×covariate interactions^27^ and GRADE methodology for appraising the certainty of the overall body of evidence for each outcome.^20 28^ We will employ funnel plots to assess potential publication bias.

### Power calculations

The primary objective of the ICS-RECODE study is to assess the interaction between ICS and BEC for the primary outcomes. Power calculations are based on eligible RCTs with confirmed access to IPD, capturing the main outcomes and at least one BEC (19 trials, assessing 39 452 eligible participants, online supplemental table S1). Based on 1000 simulations, the power to detect an interaction between ICS and BEC was >99.9% with an alpha of 5% for the 12-month rate of severe and moderate-to-severe exacerbations. BEC was simulated to have similar characteristics to the Copenhagen General Population Study^29^ and the estimated exacerbation rate was sourced from a previous post hoc analysis of three eligible RCTs.^30^ Based on these assumptions, we targeted 25% heterogeneity with a study-level random parameter.

### Patient and public involvement

Our research group, supported by the Manchester University NHS Foundation Trust Patient and Public Involvement and Engagement specialist team (Vocal), has a strong ethos for involving patients and the public in the prioritisation of research questions, study design, delivery, interpretation, dissemination and oversight. Patients have been engaged and contributed to the conceptualisation and design of this study (box 2). Moreover, table 1 summarises the planned involvement of patients and the public in our project, along with their impact to date, following the ACTIVE framework of patient, public and stakeholder involvement in systematic reviews.^24^

Box 2 **Patient engagement in the contextualisation and design of the predictors of treatment REsponse to ICS in COPD: a randomised controlled trials (RCTs) individual participant Data re-Evaluation (ICS-RECODE) study**

- Three focus groups (n=31) and eight interviews with chronic obstructive pulmonary disease (COPD) patients informed the conceptualisation and design of the ICS-RECODE study:
  - Contributed to the selection and prioritisation of outcomes and covariates (eg, exercise capacity, fatigue, sleep quality).
  - Contributed to the development of a lay abstract for the study.
- Two patient representatives (JL and AP) with lived COPD experience joined the research team as lay researchers from the outset.
- Asthma+Lung UK and the COPD Foundation reviewed and supported the proposal, recognising the significant anticipated patient benefits. Both organisations committed to facilitating patient and public involvement engagement and dissemination through their networks.
- The study aligns with the James Lind Alliance’s top priorities for COPD exacerbations: preventing exacerbations.^38^ ^34^

**Table 1:** Patient, public and stakeholder engagement in the ICS-RECODE study (”Be ACTIVE” - ACTIVE framework)

Framework construct	Answers	Details
Who is involved?	Patients, carers, healthcare providers, guideline developers, researchers, pharma representatives	Two patients are involved in the core research group and one patient in the study steering committee. A patient and carer panel (n=10–15) will offer ongoing patient input. This will be complemented by input from the Asthma+Lung UK and COPD Foundation patient panels. Independent multi-stakeholder advisory group.
How are people recruited?	Patients/carers: Open recruitment.Other stakeholders: Closed recruitment.Fixed period	Stakeholders were recruited at study onset. Patients/carers were recruited through open advertisement locally in Greater Manchester. Asthma+Lung UK and the COPD Foundation are also engaged and their patient panels/advocates will offer further patient input. Representatives from other relevant stakeholder groups were invited directly by the core research group.
When are people involved?	Prioritisation, design, conduct, analysis, reporting, dissemination.	Patients are involved in all stages of this research project: prioritisation (previous focus groups); input in the design (previous focus groups and this study’s patient advocates), conduct, analysis, reporting and dissemination. Other stakeholder groups inform the design, conduct, analysis, reporting and dissemination.
How are people involved? (approach)	Continuous involvement.	Continuous, regular and ad hoc engagement with patients, carers and other stakeholders is planned through their involvement in the core research group, study steering committee patient and carer panel and the independent advisory committee.
How are people involved? (level of engagement)	**Patients and carers:** Influencing and controlling.**Other stakeholders:** Contributing	Through their involvement in the core research group and study steering committee, patients will have a controlling role in this study. Through their involvement in the patient and carer panel, they will have an influencing role, as their recommendations will inform all aspects of our study. Through their involvement in the independent advisory group, various stakeholder groups will contribute to the study.
How are people involved (format and methods)		Two patients are members of the core research group and one patient is member of the study steering committee. These patients will be involved in all meetings. Plain English language will be used for their benefit. Two focus group meetings are planned for our patient and carers panel (n=10–15), along with ongoing engagement via focused emails, polls, dissemination and other activities. The views of other stakeholders will be sourced through regular (annual) and ad hoc (as needed) meetings of the independent advisory group.

ICS-RECODEpredictors of treatment REsponse to inhaled corticosteroids (ICS) in Chronic Obstructive pulmonary disease: randomised controlled trials individual participant Data re-Evaluation

### Research team, governance, funding

The ICS-RECODE study will be conducted by a multidisciplinary team of experts in COPD therapeutics, precision medicine interventions, biostatistics, systematic review, meta-analysis and evidence-based medicine. Additionally, our team includes two lay researchers with lived experience of COPD. A steering committee, comprising professionals and a lay member with similar expertise, will oversee the project’s progress and ensure adherence to rigorous governance standards.

An advisory group will provide independent advice and expertise. This group will include representatives from primary, secondary and tertiary care, original trial investigators and sponsors, the NICE, UK and patients. Their involvement will ensure meaningful engagement of all relevant stakeholders, enhancing the relevance and impact of our research.

This study was sponsored by Manchester University NHS Foundation Trust and was funded by the National Institute for Health and Care Research Health Technology Assessment (NIHR HTA; NIHR152516). The study protocol was developed by the investigators, independently of the sponsor, funders and data contributors.

### Protocol version history

This protocol was prospectively registered with PROSPERO prior to the initiation of any analyses. The current version was finalised during the reanalysis of the included RCTs, before performing meta-analyses. A summary of modifications made since the PROSPERO registration is found in online supplemental table S2.

### Ethics and dissemination

The Health Research Authority approved the ICS-RECODE study, exempting it from ethics review (HRA UK, Reference: 24/HRA/0460).

The findings of this study will be published in high-impact peer-reviewed journals and will be presented in national and international conferences. A lay summary of the main findings will be developed with input from our patient and public involvement and engagement group. These reports will be disseminated by our research group to the scientific community, patient organisations, policymakers and the broader public.

## Discussion

The ICS-RECODE IPD meta-analysis will compile high-quality data from 22 RCTs, encompassing >50 000 participants, to rigorously evaluate the safety, efficacy, and predictors of treatment response of ICS in COPD management. The main objective is to address the evidence gap concerning BEC as a therapeutic biomarker for guiding ICS use in COPD. Additionally, it will explore other potential treatment×covariate interactions, further informing a precision medicine approach to ICS utilisation.

Several challenges will need to be addressed throughout our analysis. Included trials may capture heterogeneous data and use heterogeneous outcomes and measurement instruments. Variability has been observed not only in the selection of patient-reported outcome tools but also in the definition of endpoints such as exacerbations (eg, event-based vs symptom-based definitions).^31^ There is no standardised approach to addressing key prognostic factors like comorbidities, which are prevalent and strongly associated with the disease outcomes.^32^ Our methods will be guided by expert, multistakeholder input and consensus, ensuring transparency throughout. Last, ICSs are characterised by well-known withdrawal effects,^25^ which might affect treatment comparisons, and, perhaps more significantly, the observed interaction effects. Sensitivity analyses have been planned to assess their impact on our findings.

BECs are widely used to guide ICS initiation and discontinuation for COPD.^13^ However, our post hoc analyses of the Inhaled Steroids in obstructive lung disease in Europe (ISOLDE)^33^ and Effect of Indacaterol Glycopyronium vs Fluticasone Salmeterol on COPD Exacerbations (FLAME)^12^ trials demonstrate that BEC is a responsive biomarker; BEC levels measured on corticosteroids differ from those off ICS. ICS suppresses BEC in 40% of COPD patients, who benefit clinically without significant side effects. Conversely, BEC rises on ICS in up to 20% of patients, who experience side effects without clinical benefits. In the remaining 40%, treatment effects and safety signals are more blunted. For these reasons, we plan rigorous assessments of BEC off and/or on ICS through the ICS-RECODE study.

Wide stakeholder engagement is an important strength that informs the design and conduct of this study and will support the dissemination of our findings. It will also help to identify unmet clinical and research needs for future research. Characteristically, patients have highlighted outcomes they consider critical that are not routinely assessed in large RCTs, such as exercise capacity, sleep quality and fatigue. These findings align with the ERS COPD exacerbations’ core outcome set that prioritised functional and quality of life outcomes not routinely assessed in RCTs.^34^,^36^ This highlighted the need for a core outcome set for maintenance management of COPD, to ensure that the views of all relevant stakeholders are captured in the selection of outcomes in future RCTs.

The main strength of the ICS-RECODE study is its comprehensive RCT IPD database, offering rigorous participant and outcome characterisation, enhanced statistical power and robust insights into treatment effects, safety and response variability. In collaboration with our partners, we strive to maximise the potential of this dataset by addressing key clinical and methodological questions. In addition to the IPD meta-analysis, we are planning to develop a (multivariable) predictive model of treatment response to ICS in COPD and to evaluate the relative burden of severe COPD exacerbations versus hospitalised episodes of pneumonia to patients with COPD receiving or not receiving ICS. In a series of Studies Within a Review methodological studies,^37^ we will assess the differences between the Cochrane RoB, RoB-2 and RoB-2 informed by IPD tools, the impact of baseline treatment withdrawal effects on findings and the impact of addressing for baseline characteristics on the trial results. Overall, our high-quality dataset and stronc collaborations with our partners should help transform the management of patients with COPD, and inform the methods used in future research.

## supplementary material

10.1136/bmjopen-2024-095541 online supplemental file 1
