# Supplementary material for: Predictors of treatment REsponse to inhaled corticosteroids (ICS) in Chronic Obstructive pulmonary disease: randomised controlled trials individual participant Data re-Evaluation–protocol of the ICS-RECODE individual participant data meta-analysis
Source: BMJ Open. 2025 Mar 5;15(3):e095541. doi: 10.1136/bmjopen-2024-095541 (PMC11883585; doi:10.1136/bmjopen-2024-095541)
Supplement: online supplemental file 1 [file bmjopen-15-3-s001.docx]

**Online Supplement**

Predictors of treatment REsponse to inhaled corticosteroids (ICS) in Chronic Obstructive pulmonary disease: randomised controlled trials individual participant Data re-Evaluation. Protocol of the ICS-RECODE individual participant data meta-analysis.

Sebastian Bate, Rebecca Fortescue, Catherine Fullwood, Matthew Sperrin, Mark Simmonds^5^, Markus Fally, Jan Hansel, Michael Miligkos, Sinduja Manohar, Emily Howlett, John Linnell, Alan Preston, the ICS-RECODE group, Ashley A Woodcock, Dave Singh, Lesley A Stewart, Jørgen Vestbo, Alexander G Mathioudakis.

**Supplementary table S1.** Main characteristics of the eligible and included studies.

| **Trial ID,**  **Trial name** | **Pharma company** | **Eligible interventions** | | | | | | **Eligible active interventions: Details** | **Eligible N**  **(Overall N)** | **Duration** | **BEC availability** | **Data access request status** |
| --- | --- | --- | --- | --- | --- | --- | --- | --- | --- | --- | --- | --- |
|  |  | **LABA+LAMA+ICS** | **LABA+LAMA** | **LABA+ICS** | **LABA** | **ICS** | **Placebo** |  |  |  |  |  |
| **NCT01313676, SUMMIT** | GSK |  |  | x | x | x | x | Fluticasone furoate (100μg) + Vilanterol (25μg)  Fluticasone furoate (100μg)  Vilanterol (25μg) | 16590  (16590) | 93 weeks (median) | No | **Approved** |
| **NCT02465567,**  **ETHOS** | AZ | x | x |  |  |  |  | Budesonide (320μg) + Glycopyrollate (18μg) + Formoterol (9.6μg)  Budesonide (160μg) + Glycopyrollate (18μg) + Formoterol (9.6μg)  Glycopyrollate (18μg) + Formoterol (9.6μg) | 6437  (8588) | 52 weeks | Yes | **Declined** |
| **NCT02164513,**  **IMPACT** | GSK | x | x |  |  |  |  | Fluticasone furoate (100μg) + Vilanterol (25μg) + Umeclidinium (62.5μg)  Vilanterol (25μg) + Umeclidinium (62.5μg) | 6221  (10355) | 52 weeks | Yes | **Approved** |
| **NCT00268216,**  **TORCH** | GSK |  |  | x | x | x | x | Fluticasone propionate (500μg) + Salmeterol (50μg)  Fluticasone propionate (500μg)  Salmeterol (50μg) | 6112  (6112) | 156 weeks | No | **Approved** |
| **NCT00975195,**  **WISDOM** | BI | x | x |  |  |  |  | Fluticasone propionate (500μg) + Salmeterol (50μg) + Tiotropium (18μg)  Salmeterol (50μg) + Tiotropium (18μg) | 2488  (2488) | 52 weeks | Yes | **Approved** |
| **NCT02727660,**  **SOPHOS** | AZ |  |  | x | x |  |  | Budesonide (320μg) + Formoterol (10μg)  Budesonide (160μg) + Formoterol (10μg)  Formoterol (10μg) | 1864  (1864) | 33.2 weeks (median) | Yes | **Declined** |
| **EudraCT 2012–004162–17,**  **EFFECT** | Mundipharma |  |  | x | x |  |  | Fluticasone (500μg) + Formoterol (20μg)  Fluticasone (250μg) + Formoterol (10μg)  Formoterol (12μg) | 1765  (1765) | 52 weeks | Yes | **Approved** |
| **NCT02766608,**  **TELOS** | AZ |  |  | x | x |  |  | Budesonide (400μg) + Formoterol (12μg)  Budesonide (320μg) + Formoterol (9μg)  Budesonide (160μg) + Formoterol (9μg)  Formoterol (9μg) | 1741  (2389) | 24 weeks | Yes | **Declined** |
| **NCT00206154,**  **SHINE** | AZ |  |  | x | x | x | x | Budesonide (320μg) + Formoterol (9μg)  Budesonide (160μg) + Formoterol (9μg)  Budesonide (400μg)  Formoterol (9μg) | 1704  (1704) | 26 weeks | Yes | **Declined** |
| **NCT01017952,**  **Dransfield et al** | GSK |  |  | x | x |  |  | Fluticasone furoate (200μg) + Vilanterol (25μg)  Fluticasone furoate (100μg) + Vilanterol (25μg)  Fluticasone furoate (50μg) + Vilanterol (25μg)  Vilanterol (25μg) | 1633  (1633) | 52 weeks | Yes | **Approved** |
| **NCT01009463,**  **Dransfield et al** | GSK |  |  | x | x |  |  | Fluticasone furoate (200μg) + Vilanterol (25μg)  Fluticasone furoate (100μg) + Vilanterol (25μg)  Fluticasone furoate (50μg) + Vilanterol (25μg)  Vilanterol (25μg) | 1622  (1622) | 52 weeks | Yes | **Approved** |
| **NCT02105974,**  **Siler et al** | GSK |  |  | x | x |  |  | Fluticasone furoate (100μg) + Vilanterol (25μg)  Vilanterol (25μg) | 1622  (1622) | 12 weeks | No | **Approved** |
| **NCT02579850,**  **TRIBUTE** | Chiesi | x | x |  |  |  |  | Beclomethasone dipropionate (87μg) + Formoterol (5μg) + Glycopyrronium (9μg)  Indacaterol (85μg) + Glycopyrronium (43μg) | 1532  (1532) | 52 weeks | Yes | **Approved** |
| **NCT00206167,**  **Rennard et al** | AZ |  |  | x | x |  |  | Budesonide (320μg) + Formoterol (9μg)  Budesonide (160μg) + Formoterol (9μg)  Formoterol (9μg) | 1483  (1964) | 52 weeks | Yes | **Declined** |
| **SFCB3024,**  **TRISTAN** | GSK |  |  | x | x | x | x | Fluticasone propionate (500μg) + Salmeterol (50μg)  Fluticasone propionate (500μg)  Salmeterol (50μg) | 1465  (1465) | 52 weeks | Yes | **Approved** |
| **NCT01069289,**  **SUMIRE** | AZ |  |  | x | x |  |  | Budesonide (320μg) + Formoterol (9μg)  Formoterol (9μg) | 1293  (1293) | 12 weeks | Yes | **Approved** |
| **NCT02497001,**  **KRONOS** | AZ | x | x |  |  |  |  | Budesonide (320μg) + Glycopyrollate (18μg) + Formoterol (9.6μg)  Glycopyrollate (18μg) + Formoterol (9.6μg) | 1267  (1902) | 24 weeks | Yes | **Declined** |
| **NCT01054885, Martinez et al** | GSK |  |  | x | x | x | x | Fluticasone furoate (200μg) + Vilanterol (25μg)  Fluticasone furoate (100μg) + Vilanterol (25μg)  Fluticasone furoate (200μg)  Fluticasone furoate (100μg)  Vilanterol (25μg) | 1224  (1224) | 52 weeks | Yes | **Approved** |
| **NCT02157935,**  **RISE** | AZ |  |  | x | x |  |  | Budesonide (320μg) + Formoterol (9μg)  Formoterol (9μg) | 1219  (1219) | 26 weeks | Yes | **Approved** |
| **NCT00419744,**  **Sharafkhaneh et al** | AZ |  |  | x | x |  |  | Budesonide (320μg) + Formoterol (9μg)  Budesonide (160μg) + Formoterol (9μg)  Formoterol (9μg) | 1219  (1219) | 52 weeks | Yes | **Approved** |
| **NCT00929851,**  **FORWARD** | Chiesi |  |  | x | x |  |  | Beclomethasone dipropionate (200μg) + Formoterol (12μg)  Formoterol (12μg) | 1186  (1186) | 48 weeks | Yes | **Approved** |
| **NCT02603393,**  **SUNSET** | Novartis | x | x |  |  |  |  | Fluticasone propionate (500μg) + Salmeterol (50μg) + Tiotropium (18μg)  Indacaterol (110μg) + Glycopyrronium (50μg) | 1053  (1053) | 24 weeks | Yes | **Approved** |
| **NCT01053988,**  **Kerwin et al** | GSK |  |  | x | x | x | x | Fluticasone furoate (100μg) + Vilanterol (25μg)  Fluticasone furoate (50μg) + Vilanterol (25μg)  Fluticasone furoate (100μg)  Vilanterol (25μg) | 1030  (1030) | 24 weeks | Yes | **Approved** |
| **D5892C00670,**  **Calverley et al** | AZ |  |  | x | x | x | x | Budesonide (320μg) + Formoterol (9μg)  Budesonide (400μg)  Formoterol (9μg) | 1022  (1022) | 52 weeks | No | **Declined** |
| **Kardos et al** | GSK |  |  | x | x |  |  | Fluticasone (500μg) + Salmeterol (50μg)  Salmeterol (50μg) | 994  (994) | 52 weeks | No | **Unavailable** |
| **D5892C00629,**  **Szafranski et al** | AZ |  |  | x | x | x | x | Budesonide (320μg) + Formoterol (9μg)  Budesonide (400μg)  Formoterol (9μg) | 812  (812) | 52 weeks | No | **Declined** |
| **NCT00115492,**  **Anzueto et al** | GSK |  |  | x | x |  |  | Fluticasone propionate (500μg) + Salmeterol (50μg)  Salmeterol (50μg) | 797  (797) | 52 weeks | No | **Approved** |
| **NCT00144911,**  **Ferguson et al** | GSK |  |  | x | x |  |  | Fluticasone propionate (500μg) + Salmeterol (50μg)  Salmeterol (50μg) | 782  (782) | 52 weeks | No | **Approved** |
| **NCT00476099,**  **Calverley et al** | Chiesi |  |  | x | x |  |  | Beclomethasone dipropionate (400μg) + Formoterol (12μg)  Beclomethasone dipropionate (200μg) + Formoterol (12μg)  Formoterol (12μg) | 718  (718) | 48 weeks | Yes | **Approved** |
| **NCT01110200,**  **Ohar et al** | GSK |  |  | x | x |  |  | Fluticasone propionate (250μg) + Salmeterol (50μg)  Salmeterol (50μg) | 639  (639) | 26 weeks | No | **Approved** |
| **NCT01555138**  **INSTEAD** | Novartis |  |  | x | x |  |  | Fluticasone (500μg) + Salmeterol (50μg)  Indacaterol (150μg) | 581  (581) | 26 weeks | Yes | **Approved** |

**Supplementary table S2.** Modifications since PROSPERO registration. Our protocol was prospectively registered with PROSPERO before commencing analyses. This version of the protocol was finalised during the re-analysis of the included RCTs but prior to conducting the meta-analyses.

| Secondary outcomes | We added three secondary outcomes: exercise capacity, fatigue, sleep quality. These were prioritised by patients during our patient focus groups. |
| --- | --- |
| Secondary outcomes: Death | We initially considered reporting death as both a dichotomous outcome and a time-to-event outcome. However, due to the limited number of deaths observed in the included trials, we deemed the dichotomous approach less informative and decided to exclude it. |
| Secondary outcomes: Serious adverse events | We were originally planning on assessing time-to-first serious adverse events and number of participants with at least one serious adverse event. However, we found these measures to be too similar and decided to replace the latter with the rate of serious adverse events. |
| Outcome assessment timepoints | We clarified the outcome assessment timepoints, that were missing from the previous version |
| Covariates: Predominant type of exacerbations | We planned to consider predominant type of exacerbations (treated with antibiotics or with systemic corticosteroids) as a covariate. However, such a covariate would have introduced post-randomisation and collider bias, while many study participants did not experience an exacerbation during follow-up or did not have a clearly predominant type. For these reasons, we decided to remove this covariate. Instead, we assessed types of exacerbations as secondary outcomes. |
| Prognostic factors: Comorbidities | In our PROSPERO registration, we indicated that "main comorbidities" would be addressed as a prognostic factor. Here, we further clarify that comorbidities will be evaluated as a count variable, representing the number of affected body systems. |
| Prognostic factors: Treatment adherence | We planned to consider treatment adherence as an additional prognostic factor. However, this would have introduced post-randomisation and collider bias. For this reason we decided to remove this variable. |
