# Supplementary material for: Predictors of treatment REsponse to inhaled corticosteroids (ICS) in Chronic Obstructive pulmonary disease: randomised controlled trials individual participant Data re-Evaluation–protocol of the ICS-RECODE individual participant data meta-analysis
Source: BMJ Open. 2025 Mar 5;15(3):e095541. doi: 10.1136/bmjopen-2024-095541 (PMC11883585; doi:10.1136/bmjopen-2024-095541)
Supplement: Supplementary file 2 [file bmjopen-2024-095541.reviewer_comments.pdf]

PEER REVIEW HISTORY

BMJ Open publishes all reviews undertaken for accepted manuscripts. Reviewers are asked to complete a checklist review form and are provided with free text boxes to elaborate on their assessment. These free text comments are reproduced below.

ARTICLE DETAILS

Title (Provisional)

Predictors of treatment REsponse to inhaled corticosteroids (ICS) in Chronic Obstructive pulmonary disease: randomised controlled trials individual participant Data re-Evaluation. Protocol of the ICS-RECODE individual participant data meta-analysis.

Authors

Bate, Sebastian; Fortescue, Rebecca; Fullwood, Catherine; Sperrin, Matthew; Simmonds, Mark; Fally, Markus; Hansel , Jan; Miligkos, Michael; Manohar, Sinduja; Howlett, Emily; Linnell, John; Preston, Alan; the ICS-RECODE group, .; Woodcock, Ashley A; Singh, Dave; Stewart, Lesley; Vestbo, Joergen; Mathioudakis, Alexander G.

VERSION 1 - REVIEW

**Reviewer**	**1**
**Name**	**Alagha, Zakaria**
**Affiliation**	**Marshall University School of Medicine**
**Date**	**28-Nov-2024**
**COI**	**None**

Thank you for the opportunity to review this protocol.

The outcomes section is thorough and well-organized, with clear definitions for the primary outcomes (moderate and severe exacerbations) and a wide range of secondary outcomes, almost all measured using established instruments. Patient-centered measures and defined follow-up periods are among its strengths along with clear well- written method section.

I would suggest addressing the following:

Clarifying whether the secondary outcomes will be considered equally or if some will be prioritized in the analysis would add focus and improve interpretability.

I would suggest including heart failure and pulmonary hypertension among the secondary outcomes.

You mentioned that all continuous outcomes will be assessed as "change from baseline" is reasonable, but the statistical procedures for dealing with these measurements should be described.

While not expressly stated, examining outcomes by demographic or clinical subgroups may yield more nuanced results.

#Mention methods (e.g., funnel plots, Egger's test) for assessing publication bias.

#Explain how the models will be internally/ externally validated.

Sensitivity analyses for key secondary outcomes could provide further insights

Discussion is well-written, and authors are aware of the limitations.

Overall, it's a well-written protocol which could address a well-developed research question.

**Reviewer**	**2**
**Name**	**Jones, Arwel**
**Affiliation**	**Monash University, Respiratory Research@Alfred**
**Date**	**12-Dec-2024**
**COI**	**None**

Apologies if the time that is taken me to get to this review has delayed you receiving a response on your manuscript - I got to it as soon as I could!

This is interesting work and I'm very pleased to see that broader project has the involvement of patients and the public, working together with the researchers. I'm not a statistical expert in Individual Participant Data meta-analysis, so there are some aspects of the data synthesis that I have not been able to review with complete certainty, but overall, the proposed approach and methods appear sound and comprehensive. I can appreciate that this evidence synthesis will have outcomes of value to the field, particularly in an era of precision medicine. Well done to you as a team for taking on this amount of work.

I have some comments on the manuscript that when addressed (in my opinion) will help to clarify/improve areas of your protocol:

Introduction/Discussion: "Blood eosinophil count (BEC) is used to guide ICS administration for COPD."; “BECs are widely used to guide ICS initiation and discontinuation for COPD.” I feel like you need a citation for each of these statements of BEC use or revise slightly to be more around suggested/recommended with an appropriate citation.

"Characteristically, inhaled corticosteroids (ICS) appear to be effective only for patients with enhanced airway eosinophilic inflammation". I appreciate the work that has been done on blood eosinophils as a marker of inhaled corticosteroid effectiveness. I notice sputum

eosinophil count is not covered at all in the treatment/covariate interactions, was that considered at all – especially when you are going to the extent of retrieving IPD?

Primary outcome: Can you clarify how you will handle data for 'moderate or severe exacerbations'? You state how you will handle ED visits. For example, what happens if a trial reports moderate exacerbations only (perhaps you know there won't be any), will you only consider data where moderate-to-severe exacerbations is the outcome defined by the study? I see that you have rate of severe exacerbations as another primary outcome but you are not interested in rate of moderate exacerbations alone?

Secondary outcome: Exacerbations: Given the primary outcomes, I assume rate of exacerbations of any severity also includes mild exacerbations (e.g. SABA use only). If that is indeed the case, please provide a definition (for the purposes of this work) for mild exacerbations, like you have done for moderate and severe.

Secondary outcomes on PROSPERO: There is some discrepancy between some of the outcomes in this manuscript and your PROSPERO record. Your manuscript reports sleep quality as an outcome but this was not stated in your PROSPERO record in February 2024. Likewise, your serious adverse events in the manuscripts reports this outcome as rate/time to first event but your PROSPERO notes a dichotomous outcome (number with at least one event and time-to-first). Mortality in PROSPERO has number of deaths and time-to-death, your manuscript has time-to-death only. Fatigue is reported in your manuscript but this was not mentioned in your PROSPERO record. Please clarify/ revise and if these are indeed changes following registration, please consider acknowledging this in your methods or discussion where appropriate.

Covariates: (ix) smoking status. Can you clarify this? I presume it is baseline smoking status? What will be considered here – former vs current?

Covariates: (x) predominant type of exacerbations (treated with antibiotics, systemic corticosteroids or both). What is meant here – is this with regards to the moderate exacerbation outcome data - not prior exacerbations?

Prognostic factors: Your PROSPERO record says 'main comorbidities' and your manuscript states 'comorbidities, defined as number of affected body systems'. Are there specific comorbidities that you are interested (e.g. cardiovascular disease) or rather is there a limit on the number of body systems? You state in the discussion that "There is no standardized approach to addressing key prognostic factors like comorbidities, that are prevalent and strongly associated with the disease outcomes31. Our methods will be guided by expert, multistakeholder input and consensus, ensuring transparency throughout" There is the potential for bias here and some may argue there is no reason why key comorbidities can't be prespecified like you have done for other factors.

Prognostic factors: Treatment adherence is not in the manuscript but was on PROSPERO. Is this no longer needed – did you consider it not to be an effect modifier? I note you have added 'those that did not receive any doses of the study treatment' to the review criteria where I don't think it was in your PROSPERO record.

Sensitivity analyses: No inclusion of ICS dose here but listed in PROSPERO – is that because you are already considering it within your treatment-co-variate interactions?

Discussion: "Characteristically, patients have highlighted outcomes they consider critical that are not routinely assessed in large RCTs, such as exercise capacity, sleep quality and fatigue. " Perhaps the changes in reporting of sleep quality and fatigue as outcomes in this manuscript relate to the timing of your patient engagement (occurred after your protocol registration)? If that is the case, it would be appropriate to mention that here for full transparency.

Hope you find the above comments useful.

VERSION 1 - AUTHOR RESPONSE

Reviewer: 1

Dr. Zakaria Alagha, Marshall University School of Medicine

Comments to the Author:

Thank you for the opportunity to review this protocol.

The outcomes section is thorough and well-organized, with clear definitions for the primary outcomes (moderate and severe exacerbations) and a wide range of secondary outcomes, almost all measured using established instruments. Patient-centered measures and defined follow-up periods are among its strengths along with clear well- written method section.

Response: We thank the reviewer for their valuable comments.

I would suggest addressing the following:

Clarifying whether the secondary outcomes will be considered equally or if some will be prioritized in the analysis would add focus and improve interpretability.

Response: We clarified that "Secondary outcomes will be treated equally, without hierarchy."

I would suggest including heart failure and pulmonary hypertension among the secondary outcomes.

Response: Unfortunately, since the follow-up of most included RCTs is limited to 1 year or less (and none formally addressed these outcomes during follow-up), we do not believe that we can properly assess these outcomes in this study. In the future, we fully intend to assess (1) myocardial infarction, and (2) major adverse cardiovascular events. However, we did not apply for permission to assess any of these outcomes in our original study data request. Therefore, we will need to re-apply for data access, to assess those outcomes (patient level data request from the sponsors – this is a time consuming process, that takes ~1 year). As a result, we cannot include those outcomes in the current study protocol.

You mentioned that all continuous outcomes will be assessed as "change from baseline" is reasonable, but the statistical procedures for dealing with these measurements should be described.

Response: This is clarified in the analysis plan: "We will use generalised regression models for analysing continuous outcomes, and adjust for the outcomes' baseline values, in addition to the predefined parameters". We deleted the phrase "All continuous outcomes will be assessed as change from baseline" that was indeed generic.

While not expressly stated, examining outcomes by demographic or clinical subgroups may yield more nuanced results.

Response: We agree that examining outcomes by demographic and clinical subgroups can provide more nuanced insights. We will assess potential subgroup effects through our planned treatment-covariate interaction analyses. In accordance with GRADE and ICEMAN guidelines, we have prospectively selected a limited number of clinically relevant covariates for evaluation to minimize potential bias.

#Mention methods (e.g., funnel plots, Egger's test) for assessing publication bias.

Response: Done – "We will employ funnel plots to assess potential publication bias".

#Explain how the models will be internally/ externally validated.

Response: This manuscript reports in detail the methodology of the IPD meta-analysis, including evaluation of treatment-covariate interactions. While we have mentioned that we plan on developing predictive models of treatment response to ICS in COPD in our discussion, the methodology is beyond the scope of this manuscript.

Sensitivity analyses for key secondary outcomes could provide further insights

Response: This IPD meta-analysis represent an extensive project. We had to carefully consider the potential benefits of additional analyses against the significant additional resources required to perform such analyses. Unfortunately, we will not be able to perform sensitivity analyses for secondary outcomes.

Discussion is well-written, and authors are aware of the limitations.

Overall, it's a well-written protocol which could address a well-developed research question.

Response: We thank the reviewer for their positive feedback.

Reviewer: 2

Dr. Arwel Jones, Monash University

Comments to the Author:

Apologies if the time that is taken me to get to this review has delayed you receiving a response on your manuscript - I got to it as soon as I could!

This is interesting work and I'm very pleased to see that broader project has the involvement of patients and the public, working together with the researchers. I'm not a statistical expert in Individual Participant Data meta-analysis, so there are some aspects of the data synthesis that I have not been able to review with complete certainty, but overall, the proposed approach and methods appear sound and comprehensive. I can appreciate that this evidence synthesis will have outcomes of value to the field, particularly in an era of precision medicine. Well done to you as a team for taking on this amount of work.

Response: We thank the reviewer for their supportive feedback and productive comments.

I have some comments on the manuscript that when addressed (in my opinion) will help to clarify/improve areas of your protocol:

Introduction/Discussion: "Blood eosinophil count (BEC) is used to guide ICS administration for COPD."; “BECs are widely used to guide ICS initiation and discontinuation for COPD.” I feel like

you need a citation for each of these statements of BEC use or revise slightly to be more around suggested/recommended with an appropriate citation.

Response: We have now added a reference to the GOLD statement.

"Characteristically, inhaled corticosteroids (ICS) appear to be effective only for patients with enhanced airway eosinophilic inflammation". I appreciate the work that has been done on blood eosinophils as a marker of inhaled corticosteroid effectiveness. I notice sputum eosinophil count is not covered at all in the treatment/covariate interactions, was that considered at all – especially when you are going to the extent of retrieving IPD?

Response: Unfortunately, sputum eosinophil count is not often assessed in the context of RCTs, due to the additional burden to patients, as well as the analytical burden. We fully agree this is a very important biomarker, but we did not include it in our analyses as we did not expect to find any relevant data (and indeed, they were only assessed in small subgroups of participants of a small number of studies).

Primary outcome: Can you clarify how you will handle data for 'moderate or severe exacerbations'? You state how you will handle ED visits. For example, what happens if a trial reports moderate exacerbations only (perhaps you know there won't be any), will you only consider data where moderate-to-severe exacerbations is the outcome defined by the study? I see that you have rate of severe exacerbations as another primary outcome but you are not interested in rate of moderate exacerbations alone?

Response: None of the included RCTs reported moderate but not severe exacerbations. However, if there were any such trials, they would not be included in the moderate/severe exacerbations outcomes.

Severe exacerbations, defined as those requiring hospitalisation, are a clinically meaningful outcome due to their significant impact on patient health, healthcare utilisation, and prognosis. In contrast, moderate exacerbations alone are less clinically valuable, as their interpretation can be ambiguous. A reduction in moderate exacerbations might reflect a true decrease in exacerbation frequency but could also indicate a worsening in severity, with more events progressing to severe exacerbations requiring hospitalisation. For these reasons, we have focused on severe and moderate/severe exacerbations as primary outcomes to ensure our findings are both clinically relevant and interpretable.

Secondary outcome: Exacerbations: Given the primary outcomes, I assume rate of exacerbations of any severity also includes mild exacerbations (e.g. SABA use only). If that is indeed the case, please provide a definition (for the purposes of this work) for mild exacerbations, like you have done for moderate and severe.

Response: Done: "Mild exacerbations are acute episodes of increased symptoms, that are beyond the normal day-to-day variation, that do not necessitate systemic treatment (antibiotics or corticosteroids) or hospital admission. We will accept patient reported mild exacerbations, as well as those identified through validated questionnaires or patient diaries".

Secondary outcomes on PROSPERO: There is some discrepancy between some of the outcomes in this manuscript and your PROSPERO record. Your manuscript reports sleep quality as an outcome but this was not stated in your PROSPERO record in February 2024. Likewise, your serious adverse events in the manuscripts reports this outcome as rate/time

to first event but your PROSPERO notes a dichotomous outcome (number with at least one event and time-to-first). Mortality in PROSPERO has number of deaths and time-to-death, your manuscript has time-to-death only. Fatigue is reported in your manuscript but this was not mentioned in your PROSPERO record. Please clarify/ revise and if these are indeed changes following registration, please consider acknowledging this in your methods or discussion where appropriate.

Response: Thank you very much for highlighting this and following discrepancies between our protocol that is registered in PROSPERO (that was designed prospectively, before starting analysing any data), and this protocol, that is more reader friendly and we hope to help with dissemination, but was not completed prior to starting any analysis. More specifically, it was finalised while we were re-analysing the included RCTs (before performing meta-analyses). We have now clarified that the prospective protocol is the PROSPERO protocol and we have described all changes in Supplementary table S2 (including all those kindly pointed out by the reviewer):

Supplementary table S2. Modifications since PROSPERO registration. Our protocol was prospectively registered with PROSPERO before commencing analyses. This version of the protocol was finalised during the re-analysis of the included RCTs but prior to conducting the meta-analyses.

Secondary outcomes	We added three secondary outcomes: exercise capacity, fatigue, sleep quality. These were prioritised by patients during our patient focus groups.
Secondary outcomes: Death	We initially considered reporting death as both a dichotomous outcome and a time-to-event outcome. However, due to the limited number of deaths observed in the included trials, we deemed the dichotomous approach less informative and decided to exclude it.
Secondary outcomes: Serious adverse events	We were originally planning on assessing time-to-first serious adverse events and number of participants with at least one serious

	adverse event. However, we found these measures to be too similar and decided to replace the latter with the rate of serious adverse events.
Outcome assessment timepoints	We clarified the outcome assessment timepoints, that were missing from the previous version
Covariates: Predominant type of exacerbations	We planned to consider predominant type of exacerbations (treated with antibiotics or with systemic corticosteroids) as a covariate. However, such a covariate would have introduced post-randomisation and collider bias, while many study participants did not experience an exacerbation during follow-up or did not have a clearly predominant type. For these reasons, we decided to remove this covariate. Instead, we assessed types of exacerbations as secondary outcomes.
Prognostic factors: Comorbidities	In our PROSPERO registration, we indicated that "main comorbidities" would be addressed as a prognostic factor. Here, we further clarify that comorbidities will be evaluated as a count variable, representing the number of affected body systems.
Prognostic factors: Treatment adherence	We planned to consider treatment adherence as an additional prognostic factor. However, this would have introduced post-randomisation and collider bias. For this reason we decided to remove this variable.

Covariates: (ix) smoking status. Can you clarify this? I presume it is baseline smoking status? What will be considered here – former vs current?

Response: Done: “(ix) smoking status at baseline (current or former)”.

Covariates: (x) predominant type of exacerbations (treated with antibiotics, systemic corticosteroids or both). What is meant here – is this with regards to the moderate exacerbation outcome data - not prior exacerbations?

Response: We have now removed this covariate as we decided it could introduce bias.

Supplementary table S2: “We planned to consider predominant type of exacerbations

(treated with antibiotics or with systemic corticosteroids) as a covariate. However, such a covariate would have introduced post-randomisation and collider bias, while many study participants did not experience an exacerbation during follow-up or did not have a clearly predominant type. For these reasons, we decided to remove this covariate. Instead, we assessed types of exacerbations as secondary outcomes”.

Prognostic factors: Your PROSPERO record says ‘main comorbidities’ and your manuscript states ’comorbidities, defined as number of affected body systems'. Are there specific comorbidities that you are interested (e.g. cardiovascular disease) or rather is there a limit on the number of body systems? You state in the discussion that “There is no standardized approach to addressing key prognostic factors like comorbidities, that are prevalent and strongly associated with the disease outcomes31. Our methods will be guided by expert, multistakeholder input and consensus, ensuring transparency throughout” There is the potential for bias here and some may argue there is no reason why key comorbidities can’t be prespecified like you have done for other factors.

Response: We have now highlighted this change in Supplementary table S2. Our approach to addressing comorbidities in this IPD meta-analysis is based on a scoping review that we performed, discussion among our multidisciplinary group and consensus. Our literature review did not find any consensus on how to approach this issue. We opted to follow a pragmatic approach here (number of body systems affected), as these were clearly and consistently reported in most included RCTs, while the quality of reporting of specific comorbidities varied extensively across the included studies and especially in older RCTs. We fully intend to explore what is the best approach for addressing comorbidities in such studies in the future.

Prognostic factors: Treatment adherence is not in the manuscript but was on PROSPERO. Is this no longer needed – did you consider it not to be an effect modifier? I note you have added ‘those that did not receive any doses of the study treatment’ to the review criteria where I don’t think it was in your PROSPERO record.

Response: We decided to remove this prognostic factor to avoid post-randomisation and collider bias. We have clarified this is a change from the originally submitted protocol (see Supplementary table S2).

Sensitivity analyses: No inclusion of ICS dose here but listed in PROSPERO – is that because you are already considering it within your treatment-co-variate interactions?

Response: We have now aligned this with the PROSPERO protocol.

Discussion: “Characteristically, patients have highlighted outcomes they consider critical that are not routinely assessed in large RCTs, such as exercise capacity, sleep quality and fatigue. ” Perhaps the changes in reporting of sleep quality and fatigue as outcomes in this manuscript relate to the timing of your patient engagement (occurred after your protocol registration)? If that is the case, it would be appropriate to mention that here for full transparency.

Response: Done – see supplementary table 2.

Hope you find the above comments useful.

Response: The reviewer’s comments were very useful – thank you.
